# VOR Gain Is Related to Compensatory Saccades in Healthy Older Adults

**DOI:** 10.3389/fnagi.2016.00150

**Published:** 2016-06-24

**Authors:** Eric R. Anson, Robin T. Bigelow, John P. Carey, Qian-Li Xue, Stephanie Studenski, Michael C. Schubert, Yuri Agrawal

**Affiliations:** ^1^Department of Otolaryngology – Head and Neck Surgery, Johns Hopkins University School of MedicineBaltimore, MD, USA; ^2^Department of Medicine, Johns Hopkins University School of MedicineBaltimore, MD, USA; ^3^Center on Aging and Health, Johns Hopkins Medical InstitutionsBaltimore, MD, USA; ^4^Longitudinal Studies Section, National Institute on AgingBaltimore, MD, USA

**Keywords:** VOR, compensatory saccades, healthy aging, head impulse test, vestibular

## Abstract

**Objective:** Vestibulo-ocular reflex (VOR) gain is well-suited for identifying rotational vestibular dysfunction, but may miss partial progressive decline in age-related vestibular function. Since compensatory saccades might provide an alternative method for identifying subtle vestibular decline, we describe the relationship between VOR gain and compensatory saccades in healthy older adults.

**Methods**: Horizontal VOR gain was measured in 243 subjects age 60 and older from the Baltimore Longitudinal Study of Aging using video head impulse testing (HIT). Saccades in each HIT were identified as either “compensatory” or “compensatory back-up,” i.e., same or opposite direction as the VOR response respectively. Saccades were also classified as “covert” (occurring during head movement) and “overt” (occurring after head movement). The relationship between VOR gain and percentage of HITs with saccades, as well as the relationship between VOR gain and saccade latency and amplitude, were evaluated using regression analyses adjusting for age, gender, and race.

**Results:** In adjusted analyses, the percentage of HITs with compensatory saccades increased 4.5% for every 0.1 *decrease* in VOR gain (*p* < 0.0001). Overt compensatory saccade amplitude decreased 0.6° (*p* < 0.005) and latency increased 90 ms (*p* < 0.001) for every 0.1 increase in VOR gain. Covert back-up compensatory saccade amplitude increased 0.4° for every 0.1 increase in VOR gain.

**Conclusion:** We observed significant relationships between VOR gain and compensatory saccades in healthy older adults. Lower VOR gain was associated with larger amplitude, shorter latency compensatory saccades. Compensatory saccades reflect underlying rotational vestibular hypofunction, and may be particularly useful at identifying partial vestibular deficits as occur in aging adults.

## Introduction

The prevalence of vestibular dysfunction in older adults has been estimated at 30–35% (Neuhauser et al., 2008; Agrawal et al., 2009). The number of vestibular hair cells decreases with increasing age, independent of vestibular disease (Lopez et al., 2005). Vestibulo-ocular reflex (VOR) gain is a frequently used physiologic measure of vestibular function, and has been shown to decline with increased age (Li et al., 2015; McGarvie et al., 2015). The VOR gain is the amount of eye rotation relative to the amount of head rotation. When tested in the light with a visible fixation point VOR gain should be near unity (1.0), i.e., an equal and opposite eye rotation generated in response to a head rotation facilitating stable gaze. A VOR gain of <0.68 has been proposed as a cut-point between normal and abnormally low VOR gain (MacDougall et al., 2009). However, this threshold may not capture progressive vestibular loss among healthy older adults who have less pronounced VOR gain deficits (Li et al., 2015; McGarvie et al., 2015).

Compensatory saccades occur to compensate for a deficient VOR and the resulting gaze instability. The detection and quantification of these saccades has been greatly facilitated in recent years by the development of video head impulse testing (vHIT) technology permitting quantitative bedside assessment of rotational vestibular function (Weber et al., 2008; Blödow et al., 2013). Very few studies have systematically characterized compensatory saccades that occur in individuals with vestibular loss, particularly individuals with partial vestibular loss as occurs with healthy aging. The presence of saccades, saccade latency, and/or saccade amplitude may be informative about underlying VOR function, particularly in older adults with equivocal VOR gain values.

In this study we characterize compensatory saccades that occur among healthy older adults from the Baltimore Longitudinal Study of Aging (BLSA), and specifically examine the relationship between VOR gain and saccades in this cohort.

## Materials and Methods

### Participants

The BLSA is an ongoing prospective cohort study initiated by the National Institute on Aging (NIA) in 1958. Subjects are community-dwelling participants aged 20–103 who undergo a standardized array of tests over 3 days every 1–4 years at the NIA. This study evaluated a cross-sectional sample of all BLSA participants between June 2014 and April 2015 age 60 and older. During this time period 243 participants completed vHIT testing. All participants provided written informed consent, and the BLSA study protocol was approved by the Institutional Review Board at Harbor Hospital. Participants were asked to identify their race from the following options: White, Black or African Americans, Asian, American Indian or Alaska Native, Native Hawaiian or Other Pacific Islander, “Two or More Races,” “Don’t Know,” or “Refused.” Race-ethnicity was grouped as “white,” “black,” and “other” as the majority of participants were either white or black.

### Video Head Impulse Testing

Vestibular function was measured for the horizontal VOR using vHIT. Methods to measure horizontal semicircular canal function have been published previously and validated in older adults (Bartl et al., 2009; MacDougall et al., 2009; Agrawal et al., 2014). In brief, participants wore the EyeSeeCam video-oculography system, a lightweight goggle frame with a built in camera to record right eye movements and an accelerometer to record head movement at a sampling frequency of 220 Hz (Interacoustics USA, Eden Prairie, MN, USA). Participants sat approximately 1.25 m from a visual fixation target on the wall. Trained examiners tilted the participant’s head 30° below horizontal to bring the horizontal semicircular canal into the plane of head rotation and then performed 10–15 small amplitude (15–20°) head impulses to the right and left, with peak velocity typically from 150 to 200°/s.

**FIGURE 1:**
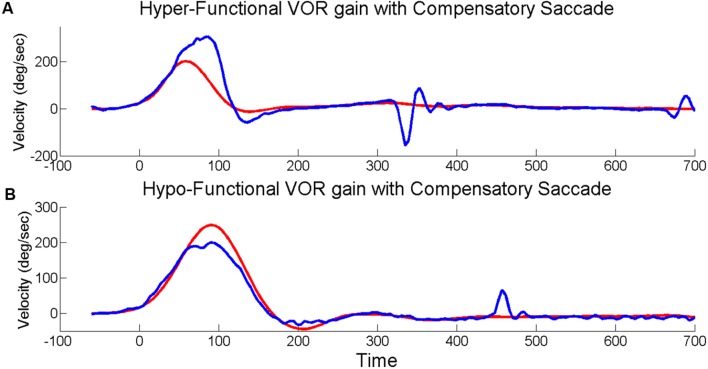
**Head impulses with saccades**. Exemplar vHITs showing the presence of an isolated back-up compensatory saccade with hyper-functional VOR gain **(A)** and compensatory saccade with hypo-functional VOR gain **(B)**. Eye velocity (blue) has been inverted and shown superimposed on head velocity (red).

The EyeSeeCam software provides up-sampled video head impulse testing (HIT) data at 1000 Hz in the exported MATLAB data file which we used for subsequent *post hoc* analyses. During *post hoc* analysis, experienced evaluators examined individual head impulse traces using custom software (MATLAB, MathWorks) and rejected head impulses that had pupil tracking artifact during the head impulse or incorrectly performed HITs (i.e., low peak head velocity, excessive head recoil, or overshoot; Mantokoudis et al., 2015b). Horizontal VOR gain was calculated as the ratio of the area under the eye velocity curve over the area under the head velocity curve from the onset of the head impulse until head velocity returned to zero (MacDougall et al., 2013). Saccades were initially identified by an automatic detection algorithm based on eye accelerations greater than 4000°/sˆ2 and visually verified by experienced examiners. Saccades in each HIT were identified as either (1) only “compensatory,” meaning same direction as the VOR; or (2) only “compensatory back-up,” meaning opposite direction as the VOR, so named based on the idea that individuals whose eye movements exceeded the head movement (i.e., the VOR overshot the target) had to make a back-up corrective saccade, see **Figure 1**. The total number of HITs to each side (right and left) was used to determine the percentage of HITs with saccades. HITs that included both compensatory and compensatory back-up saccades (∼10% of HITs), as well as HITs without any saccades (∼65% of HITs) were excluded from analyses relating VOR gain to amplitude and latency of the first saccade.

To exclude volitional saccades (i.e., saccades not generated reflexively to compensate for the VOR) we only analyzed saccades when the saccade latency was between 25 and 503 ms after HIT onset based on head velocity >20°/s (Glasauer et al., 2004). We chose 25 ms as the earliest that saccades may be present based on previously reported ranges for both active and passive HIT (Tian et al., 2007). To determine the latest time period for non-volitional reflexive saccades we first estimated the population average HIT duration from our sample with bootstrapping. We estimated 1000 new populations allowing for resampling, with 500 nested re-samples for variance estimates. This resulted in a population average HIT duration of 253 ms. We identified 250 ms after the HIT ended as the criteria after which saccades could not be reliably attributed to the HIT stimulus based on reported values for volitional saccade latencies in older adults (Abel et al., 1983; Warren et al., 2013). The percentage of HITs with only compensatory saccades and only back-up compensatory saccades was determined for each ear (right and left HITs). Saccade latency (time from the onset of the HIT until the onset of the saccade) and amplitude for the first compensatory saccade was also determined for each HIT with a saccade.

### Data Analysis

We combined rightward and leftward HITs for a total sample of 423 ears for analysis of the percentage of HITs with saccades representing 213 participants age 60 and older with saccades during HITs. Analysis of *covert* saccade latency and amplitude was based on individual HITs with compensatory saccades (*n* = 313, representing 108 participants) or back-up saccades (*n* = 252, representing 87 participants), with accounting for clustering by individual. Analysis of *overt* saccade latency and amplitude was based on individual HITs with compensatory saccades (*n* = 1675, representing 195 participants) or back-up saccades (*n* = 180, representing 88 participants), with accounting for clustering by individual. The following analyses were performed separately for HITs with only compensatory or only back-up compensatory saccades. One way ANOVA was performed to determine whether there is a difference in the percentage of compensatory and back-up compensatory saccades for each participant based on VOR gain categories (three levels: supra-unity [>1.1], normal [0.9–1.1], or low [<0.9]). Linear random effects models were used for the following analyses. First we assessed the association between percentage of HITs with compensatory and back-up compensatory saccades and VOR gain, without distinguishing between overt and covert saccades. Next we evaluated the association between latency of the first saccade and VOR gain separately for overt and covert saccades. Finally we assessed the association between amplitude of the first saccade and VOR gain separately for overt and covert saccades. We included the subject-specific mean HIT parameters (i.e., VOR gain, saccade latency, amplitude) as random effects to account for the within-subject correlation between the repeated HITs. This results in valid statistical inference for the fixed effects. We estimated the model parameters via maximum likelihood using xtmixed in STATA. All mixed effects regression models were adjusted for age, gender and race. To correct for the 10 planned comparisons an adjusted α = 0.005 was used. Separate from the planned analyses, we repeated the regression analyses with a more conservative approach by excluding HITs with VOR gain >1.2 which may represent a technical issue (Mantokoudis et al., 2015b). We also evaluated a reduced time window for acceptable saccade latencies of 170 ms after the head velocity returned to zero (Schubert et al., 2010).

## Results

**FIGURE 2:**
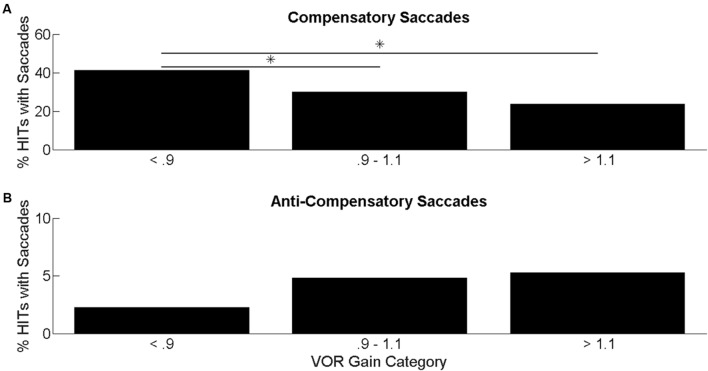
**Percentage of head impulses with saccades**. Average percentage of HITs with compensatory saccades **(A)** and back-up saccades **(B)** categorized by VOR gain. ANOVA results comparing the mean difference in percentage of HITs with saccades categorized by VOR gain. Significant differences at *p* < 0.05 are indicated by an ^∗^.

The mean age of participants was 75.9 [standard deviation (SD): 8.2, range: 60–93], and 48% of the participants were male. 63% of the participants were white, 21% of the participants were black, and the remaining 16% were classified as other. The mean (SD) VOR gain for the sample was 0.99 (0.15). The distribution of VOR gain values within the BLSA participants was as follows: 89 participants had VOR gain ≤ 0.9 (“low”); 318 participants had VOR gain between 0.9 and 1.1 (“normal”); 79 participants had VOR gain ≥ 1.1 (“super-unity”). The average percentage of HITs with compensatory or back-up compensatory saccades by VOR gain categories is shown in **Figure 2**. The percentage of HITs with compensatory saccades decreased from 40.5% in the low VOR gain category to 19.2% in the super-unity VOR gain category. In ANOVA analyses, individuals with lower VOR gain had significantly higher percentage of compensatory saccades during HITs compared to individuals with normal or supra-unity gains (**Figure 2A**). The percentage of HITs with back-up compensatory saccades increased from 2.2% in the low VOR gain category to 5.1% in the super-unity VOR gain category. In ANOVA analyses, there were no significant group differences in the percentage of back-up compensatory saccades (**Figure 2B**).

In mixed linear regression analyses, there was a significant association between lower VOR gain and higher percentage of HITs with compensatory saccades (β = -40.97, *p* < 0.001) after controlling for age, gender, and race (**Table 1**). This can be interpreted as a 4.1% increase in the percentage of HITs with compensatory saccades for every 0.1 decrease in VOR gain. There was no significant relationship between VOR gain and percentage of back-up compensatory saccades. Men had a small but significantly higher percentage of back-up compensatory saccades compared to women (β = 3.37, *p* < 0.001), after accounting for age, race, and VOR gain. We performed the same analysis after excluding the individuals with VOR gain < = 0.68 (*n* = 7 or 2% of the sample), with no change in the results (data not shown).

**Table 1:** Relationship between VOR gain and percentage of HITs with saccades.

		% of HITs with saccades		
Saccade type	Predictor variables	β	*P*	95% CI
Compensatory	Age	0.18	0.187	[**-**0.09, 0.44]
(*n* = 423 ears, 213 individuals)	**VOR gain**	**-40.97**	**0.000^∗^**	**[-54.5, -27.5]**
	Race			
	White	Ref	Ref	Ref
	Black	**-**1.22	0.646	[**-**6.4, 4.0]
	Other	2.54	0.413	[**-**3.5, 8.6]
	Gender			
	Female	Ref	Ref	Ref
	Male	3.4	0.118	[**-**0.9, 7.7]
Back-up compensatory	Age	**-**0.07	0.214	[**-**0.18, 0.04]
(*n* = 423 ears, 213 individuals)	VOR gain	2.75	0.346	[**-**2.97, 8.47]
	Race			
	White	Ref	Ref	Ref


	Black	**-**2.36	0.035	[**-**4.55, **-**0.17]


	Other	1.98	0.113	[**-**0.59, 4.54]


	Gender			
	Female	ref	Ref	ref


	**Male**	**3.37**	**0.000^∗^**	**[1.57, 5.17]**

*Significant results are bolded and indicated by ^∗^*.

### Covert Saccades

The mean (SD) latency for the first compensatory saccades was 83.5 ms (47.8 ms). The mean (SD) latency for the first compensatory back-up saccade was 98.6 ms (29.0 ms).

**FIGURE 3:**
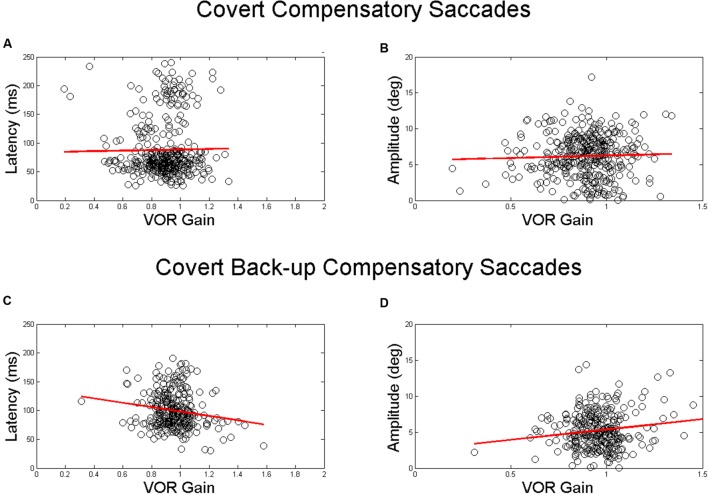
**Relationship between VOR gain and first covert saccades**. Relationship between VOR gain and covert saccade latency **(A,C)** and covert saccade amplitude **(B,D)**. The top plots demonstrate the relationship for compensatory saccades, while the lower plots show the relationship for back-up saccades. The red line in each plot indicates the linear best fit for the relationship. Each point represents a single HIT.

There were no significant associations between VOR gain and saccade latency or amplitude for covert compensatory saccades. There was a significant associations between VOR gain and saccade amplitude for covert back-up saccades (β = 3.6, *p* = 0.002), after accounting for age, race, and gender (**Table 2** and **Figure 3**). That is, there was a 0.4° increase in the amplitude of back-up compensatory saccades for each 0.1 increase in VOR gain. The mean (SD) amplitude for the first compensatory covert saccade was 6.4 (2.8) degrees. The mean (SD) amplitude for the first compensatory back-up covert saccade was 5.4 (2.6) degrees.

**Table 2:** Relationship between VOR gain and covert saccades.

		Saccade latency			Saccade amplitude		
Saccade type	Predictor variables	β	*p*	95% CI	β	*p*	95% CI
Compensatory	Age	0.43	0.464	[-0.73, 1.59]	-0.01	0.636	[0.07, 0.04]
(*n* = 313 HITs, 108 individuals)	VOR gain	32.6	0.077	[-3.53, 68.7]	-0.25	0.837	[-2.60, 2.11]
	Race						
	White	Ref	Ref	Ref	Ref	Ref	Ref
	Black	-31.28	0.017	[-56.92, -5.65]	1.09	0.088	[-0.16, 2.34]
	Other	-10.02	0.451	[-36.08, 16.03]	0.42	0.511	[-0.83, 1.67]
	Gender						
	Female	Ref	Ref	Ref	Ref	Ref	Ref
	Male	-23.2	0.023	[-43.25, -3.15]	0.60	0.226	[-0.37, 1.58]
Back-up compensatory	Age	-0.08	0.781	[-0.63, 0.47]	0.03	0.101	[-0.01, 0.08]
(*n* = 252 HITs, 87 individuals)	VOR gain	-22.55	0.089	[-48.50, 3.41]	**3.6**	**0.002^∗^**	**[1.30, 5.90]**
	Race						
	White	Ref	Ref	Ref	Ref	Ref	Ref
	Black	-13.09	0.077	[-27.61, 1.43]	0.29	0.634	[-0.91, 1.49]
	Other	3.14	0.619	[-9.26, 15.54 ]	0.52	0.265	[-0.39, 1.44]
	Gender						
	Female	Ref	Ref	Ref	Ref	Ref	Ref
	Male	-11.19	0.028	[-21.20, -1.18]	0.61	0.126	[0.17, 1.39]

*Relationship between VOR gain and (1) saccade latency, and (2) saccade amplitude for compensatory and back-up saccades. Significant results are bolded and indicated by ^∗^*.

### Overt Saccades

**FIGURE 4:**
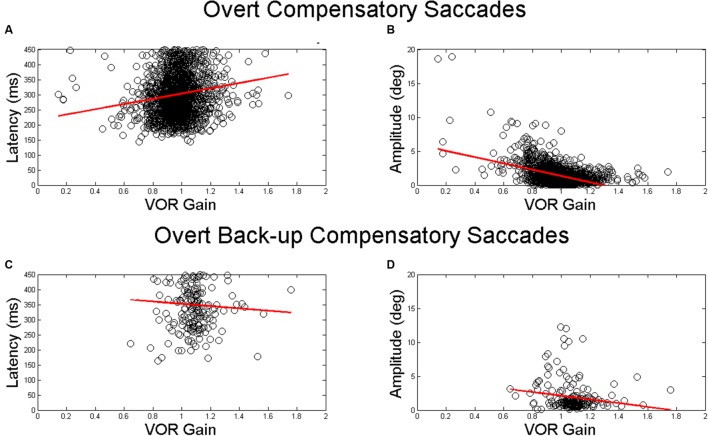
**Relationship between VOR gain and first overt saccades**. Relationship between VOR gain and overt saccade latency **(A,C)** and overt saccade amplitude **(B,D)**. The top plots demonstrate the relationship for compensatory saccades, while the lower plots show the relationship for back-up saccades. The red line in each plot indicates the linear best fit for the relationship. Each point represents a single HIT.

The mean (SD) latency for the first compensatory saccades was 302.7 ms (74.5 ms). The mean (SD) latency for the first compensatory back-up saccade was 350.0 ms (81.3 ms). There was a significant associations between VOR gain and overt saccade latency only for compensatory saccades (β = 91.08, *p* < 0.001) in adjusted analyses (**Table 3** and **Figures 4A,C**). The mean (SD) amplitude for the first compensatory saccades was 1.5 (1.4) degrees. Higher VOR gain was significantly associated with smaller first compensatory saccades (β = -3.57, *p* < 0.001) in adjusted analyses, see **Table 3** and **Figure 4B**. For every 0.1 increase in VOR gain, compensatory saccade amplitude decreased by 0.4°. There was a small but significant association between age and amplitude of compensatory saccades (β = 0.03, *p* < 0.001). The mean (SD) amplitude for the first compensatory back-up saccades was 1.9 (2.2) degrees. There were no significant associations between VOR gain and overt compensatory back-up saccade amplitude (**Table 3** and **Figure 4D**).

**Table 3:** Relationship between VOR gain and overt saccades.

		Saccade latency			Saccade amplitude		
Saccade type	Predictor variables	β	*p*	95% CI	β	*p*	95% CI
Compensatory	Age	0.22	0.651	[-0.72, 1.16]	**0.03**	**0.000^∗^**	**[0.02, 0.05]**
(*n* = 1675 HITs, 195 individuals)	VOR gain	**91.08**	**0.000^∗^**	**[58.26, 123.91]**	-**3.57**	**0.000^∗^**	**[**-**4.07,**-**3.07]**
	Race						
	White	Ref	Ref	Ref	Ref	Ref	Ref
	Black	0.76	0.935	[-17.58, 19.10]	0.04	0.789	[-0.26, 0.34]
	Other	-4.31	0.694	[-25.80, 17.19]	0.17	0.337	[-0.18, 0.53]
	Gender						
	Female	Ref	Ref	Ref	Ref	Ref	Ref
	Male	1.53	0.843	[-13.62, 16.69]	0.04	0.731	[-0.29, 0.21]
Back-up compensatory	Age	1.00	0.349	[-1.10, 3.10]	0.02	0.581	[-0.05, 0.08]
(*n* = 180 HITs, 88 individuals)	VOR gain	-14.22	0.776	[-112.12, 83.66]	0.90	0.341	[-0.95, 2.74]
	Race						
	White	Ref	Ref	Ref	Ref	Ref	Ref
	Black	6.51	0.744	[-32.57, 45.59]	0.08	0.898	[-1.10, 1.25]
	Other	13.77	0.552	[-31.67, 59.22 ]	-0.12	0.860	[-1.48, 1.24]
	Gender						
	Female	Ref	Ref	Ref	Ref	Ref	Ref
	Male	-29.05	0.067	[-0.07, -1.98]	0.46	0.348	[-0.49, 1.40]

*Relationship between VOR gain and (1) saccade latency, and (2) saccade amplitude for compensatory and back-up saccades. Significant results are bolded and indicated by ^∗^*.

We performed the same analysis after excluding the individuals with VOR gain < = 0.68 (*n* = 56 or 3.6% of HITs with compensatory saccades, *n* = 5 or 1.5% of HITs with back-up saccades), with no change in the results (data not shown). In preliminary analyses, we also included HIT duration and peak HIT velocity as proxies for HIT amplitude, but they were not significant predictors in the regressions; therefore we did not include them in the final model (data not shown).

We replicated our regression analyses on a restricted data set limiting VOR gain to < = 1.2 and limiting latency to 170 ms following HIT duration (Schubert et al., 2010). These results are presented in Supplementary Tables S1–S3. Sample sizes were reduced in this restricted data set of covert saccades (from 108 to 105 and 87 to 85 for compensatory and back-up saccades respectively). Sample sizes were reduced in this restricted data set of overt saccades (from 195 to 175 and 88 to 45 for compensatory and back-up saccades respectively). Although the pattern of relationships between VOR gain and saccade parameters was preserved, two associations were no longer significant. The association between VOR gain and amplitude of covert back-up saccades became non-significant (from β = 3.6, *p* = 0.002 to β = 2.29, *p* = 0.112). Additionally, the association between VOR gain and overt compensatory saccade latency became non-significant (from β = 91.08, *p* < 0.0001 to β = 21.24, *p* = 0.121). We additionally examined the relationship between saccade amplitude and the difference of the eye and head position (calculated as the area under the curve) at the end of the head impulse. Those results are the same as the results we report for VOR gain (data not shown).

## Discussion

In this study we characterized the specific relationships between compensatory saccades and VOR gain in a large cohort of healthy older adults. We observed that individuals were more likely to make overt compensatory saccades that were of higher amplitude and shorter latency the lower their VOR gain. Interestingly, we also found that individuals with high super-unity VOR gains were more likely to make back-up compensatory saccades. It should be noted that the majority of our participants had a “clinically normal” VOR gain, i.e., greater than a value of 0.68 (MacDougall et al., 2009). Our data demonstrate that the relationship between VOR gain and compensatory saccade metrics of latency and amplitude differs between overt and covert saccades. Compensatory saccade amplitude and latency scale according to VOR gain for overt saccades and back-up compensatory saccade amplitude scales with VOR gain for only for covert saccades. Given the observed relationships between VOR gain and saccade metrics, our data suggest that compensatory saccades capture abnormal gaze stability which may not be adequately characterized by VOR gain.

Compensatory saccades are an oculomotor gaze-stabilizing strategy to compensate for a deficient VOR, and are the key pathological sign of the clinical head impulse test (Halmagyi and Curthoys, 1988). Recent advances in quantitative VOR testing using vHIT have permitted more precise measurement of compensatory saccade characteristics in large samples (MacDougall and Curthoys, 2012). We observed in a cohort of healthy older adults that compensatory saccades were present in 20–40% of HITs, depending on the function of VOR gain. Individuals with lower VOR gain made a greater number of larger amplitude and shorter latency compensatory saccades, likely in response to increased retinal slip and a greater gaze position error. Importantly, even if not all HITs had a compensatory saccade, the compensatory saccades when present were associated with underlying VOR gain.

Our study extends several prior reports of the relationship between VOR gain and compensatory saccades. A prior study observed that covert, though not overt, saccade amplitude and latency tracked with VOR gain (Tian et al., 2000). In our study, we observed that primarily overt saccades metrics tracked with VOR gain. Several methodological differences may limit comparison between these two studies: Tian et al. (2000) rotated individuals in a chair in a dark room and the target was extinguished during rotation, while we applied passive HITs in a dimly lit room and our target was constantly visible. Tian et al. (2000) did not analyze saccades after the end of the rotation and thus it is unknown whether their subjects would have generated overt saccades whose latency and amplitude were related to the VOR gain. The difference between our current covert saccade results and those reported by Tian et al. (2000) may also depend on differences between the VOR response and the visually augmented VOR response used in our study (Tian et al., 2000). A case report from an individual with vestibular loss reported a scaling back of compensatory saccades as VOR function returned (Schubert et al., 2006). In this case, latency and amplitude of the compensatory saccades both reduced as VOR function recovered. Our study extends these findings at a population level to older adults, in whom vestibular losses due to healthy aging are typically partial (Paige, 1992). It has been argued that the timing and amplitude of saccades changes over time, an effect of learning (Lee et al., 2014; Mantokoudis et al., 2015a). In this cohort the saccade latencies all demonstrate high variability. If the participants included in this study represent various stages of adaptation to either age related vestibular dysfunction or previous partly or fully compensated peripheral vestibular dysfunction, then high variability in the latency across individuals is not unexpected. Future longitudinal studies are needed to determine whether saccades following HITs get smaller and happen earlier for older adults over time.

With the recent advent of vHIT testing, it has become evident that individuals with no history of vestibular complaints have a wide range of VOR gain values. Normative data suggest that the lower cutoff value for normal VOR gain should be 0.68 (MacDougall et al., 2009). This surprisingly low value may make it difficult to distinguish partial VOR gain deficits due to aging or vestibular pathology from normal (Li et al., 2015; McGarvie et al., 2015). Overt compensatory saccade amplitude may provide an alternative measure of the compensatory quality of the VOR because these saccades presumably reflect the perceived *inadequacy* of the VOR to prevent retinal velocity or position errors. Thus, they not only reflect the status of the peripheral end organs but also more widely the suitability of the VOR to the task of perceptual visual stabilization. Analysis of compensatory saccades may augment VOR gain by more completely characterizing overall gaze stabilization for older adults. However, based on these data an analysis only using saccade amplitude would require a prohibitive number of HITs (>100) to have the power to detect the small changes in saccade amplitude associated with incremental steps in VOR gain. Further studies are needed to determine whether VOR gain or compensatory saccade metrics better predict age related vestibular dysfunction and functional outcomes such as balance and gait.

This study is among the first to characterize in detail compensatory back-up saccades in healthy older adults. First, we found that the 2–5% prevalence of back-up saccades was 10-fold lower than the prevalence of compensatory saccades, but fivefold larger than that reported for studies of younger adults (Luis et al., 2016). Moreover, we observed that compensatory back-up saccades are significantly more likely to occur in individuals with a hyper-functioning VOR. A hyper-functioning VOR has been postulated to occur due to dysfunction of the cerebellum, which is involved in VOR calibration. Indeed, back-up saccades have been observed in patients with cerebellar degeneration (Kheradmand and Zee, 2011). A hyper-functional VOR may also result from the use of magnifying spectacles for the correction of presbyopia (Cannon et al., 1985; Li et al., 2015). Super-unity VOR gains have also been interpreted as an artifact, potentially resulting from vHIT goggle slippage and pupil-tracking error. However, the association demonstrated here between super-unity gain and the amplitude of covert back-up compensatory saccades suggests that the high gains may reflect a hyper-functional VOR requiring a back-up corrective eye movement. Several methods have been suggested to customize the fit of vHIT systems in order to reduce goggle slippage and potentially mitigate some of the excessively high VOR gains. In pilot testing we explored the use of dental putty under the goggle frame as suggested by Versino et al. (2014); however, we were unable to efficiently standardize this procedure in a high-throughput population cohort setting. Further studies are needed to establish the need and a feasible method for improving goggle stability. Black individuals had some differences in percentage of back up saccades and latency of covert saccades compared to white individuals that were not robust to adjustments for multiple comparisons. Future research should determine whether these results are influenced by pupil artifact due to darker iris pigmentation. It has recently been suggested that anti-compensatory saccades are related to spontaneous nystagmus or an imbalance in peripheral vestibular tone (Heuberger et al., 2014; Luis et al., 2016). We observed back-up saccades in the context of super-unity gains, and the back-up saccades reported in this study were not followed by compensatory saccades. This suggests that the back-up saccades were related to inadequate gaze stability and not random or erroneous saccades. However, we cannot rule out the possibility that spontaneous nystagmus contributed to the occurrence of back-up saccades reported here.

### Limitations

These data are cross-sectional and cannot be used to support causal inferences between changes in VOR gain and changes in compensatory saccade metrics. Further work will be needed to evaluate the time course of VOR gain and the evolution of compensatory saccades in healthy older adults. For the primary analysis, we accepted HITs within a relatively long (503 ms) time window from the onset of the HIT and with supra-unity gains. There is no consensus in the literature regarding the appropriate latency window for compensatory saccades to high-frequency head impulses in older adults, and we made the decision to choose 503 ms based on published volitional saccade latencies to a single cued visual target of magnitude similar to the approximate head rotation in older adults. Similarly, there is not a consensus about the interpretation of super-unity gains; which have been attributed to adaptations to spectacle correction, cerebellar disinhibition, or technical artifact (Cannon et al., 1985; Kheradmand and Zee, 2011; Li et al., 2015; Mantokoudis et al., 2015b; Matiño-Soler et al., 2015). Latencies for vestibular catch up saccades in passive rotational testing for individuals with known vestibular dysfunction occur much faster than the 503 ms cut off we imposed (Tian et al., 2000). When we limited our analysis to saccades within 170 ms following HIT and to VOR gains < = 1.2, a subset of the associations became non-significant. While this may have been a sample size and power issue, further work will be needed to elucidate the latency range of post-HIT vestibular saccades in older adults, and the significance of super-unity gains.

We also only considered horizontal VOR gain in this study, given time limitations of testing all canal planes and technical limitations with current vHIT devices in reliably measuring vertical canal VOR gain. Future studies will need to establish whether these relationships hold for the vertical canals as well. We considered amplitude of the first saccade and not peak velocity as the amplitude is more clinically interpretable and is related to the clinical HIT. Future work is forthcoming exploring the relationship of these saccades to determine if there is a main sequence for all vestibular mediated corrective saccades. Finally, to simplify our analysis and to ensure we were capturing saccades of vestibular origin, we only considered HITs resulting in either isolated compensatory or back-up compensatory saccades and only considered the first saccade. A number of HITs resulted in mixed types of saccades, and their significance will require future characterization and study.

## Conclusion

We observed that compensatory saccade metrics including saccade amplitude and latency significantly track VOR gain in a cohort of healthy older adults. In the setting of partial vestibular deficits where individuals would be classified as having normal VOR gain (i.e., VOR gain > 0.68), quantitative analysis of compensatory saccades may provide an additional characterization of age related changes to rotational vestibular function that functionally impact overall gaze stability.

## Author Contributions

EA, RB collected the data. EA, Q-LX, YA drafted the manuscript. EA, Q-LX, YA conducted the statistical analysis and interpreted the statistical analysis. EA, RB, JC, MS, SS, YA interpreted the data and critically edited the manuscript. YA designed the experiment. All authors approve the submitted version of the manuscript and are accountable for the accuracy and integrity of the work.

## Conflict of Interest Statement

The authors declare that the research was conducted in the absence of any commercial or financial relationships that could be construed as a potential conflict of interest.
