# Supplementary material for: VOR Gain Is Related to Compensatory Saccades in Healthy Older Adults
Source: Front Aging Neurosci. 2016 Jun 24;8:150. doi: 10.3389/fnagi.2016.00150 (PMC4919329; doi:10.3389/fnagi.2016.00150)
Supplement: Supplementary file 1 [file Data_Sheet_1.DOCX]

Supplementary Material

VOR gain is related to compensatory saccades in healthy older adults

ER Anson1*, RT Bigelow1, JP Carey1, Q Xue2,3, S Studenski4, MC Schubert1, Y Agrawal1

*** Correspondence:** Eric Anson: eanson1@jhmi.edu

# Supplementary Figures and Tables

**Table 1. Relationship between VOR gain and percentage of HITs with saccades after limiting VOR gain < 1.2 and overt saccade latency to 170ms following the end of the HIT.** Significant results indicated by *.

|  |  | % of HITs with Saccades | | |
| --- | --- | --- | --- | --- |
| Saccade Type | Predictor Variables | β | p | 95% CI |
| Compensatory | Age | 0.16 | 0.271 | [-0.12, 0.44] |
|  | VOR gain | -44.3 | 0.000* | [-61.31, -27.34] |
|  | Race |  |  | [-0.12, 0.44] |
|  | White | ref | ref | ref |
|  | Black | -0.97 | 0.731 | [-6.50, 4.56] |
|  | Other | 2.88 | 0.367 | [-3.38, 9.14] |
|  | Gender |  |  | [-0.12, 0.44] |
|  | Female | ref | ref | ref |
|  | Male | 3.67 | 0.107 | [-0.80, 8.13] |
| Back-up compensatory | Age | -0.08 | 0.172 | [-0.20, 0.04] |
|  | VOR gain | 2.51 | 0.491 | [-4.63, 9.65] |
|  | Race |  |  | [-0.12, 0.44] |
|  | White | ref | ref | ref |
|  | Black | -2.11 | 0.072 | [-4.41, 0.19] |
|  | Other | 2.10 | 0.133 | [-0.50, 4.70] |
|  | Gender |  |  |  |
|  | Female | ref | ref | ref |
|  | Male | 3.27 | 0.001 | [1.42, 5.13] |

**Table 2. Relationship between VOR gain and covert saccades after limiting VOR gain < 1.2 and overt saccade latency to 170ms following the end of the HIT.** Relationship between VOR gain and 1) Saccade Latency, and 2) Saccade Amplitude for compensatory and back-up saccades. Significant results indicated by *.

|  |  |  | Saccade Latency | | |  | Saccade Amplitude | | |
| --- | --- | --- | --- | --- | --- | --- | --- | --- | --- |
| Saccade Type | Predictor Variables |  | β | p | 95% CI |  | β | p | 95% CI |
| Compensatory | Age |  | 0.47 | 0.412 | [-0.65, 1.60] |  | -0.02 | 0.395 | [0.08, 0.03] |
| (n = 307 HITs, 105 individuals) | VOR gain |  | 17.42 | 0.367 | [-20.4, 55.2] |  | -0.43 | 0.732 | [-2.92, 2.05] |
|  | Race |  |  |  |  |  |  |  |  |
|  | White |  | ref | ref | ref |  | ref | ref | ref |
|  | Black |  | -28.78 | 0.023 | [-53.6, -4.01] |  | 1.07 | 0.088 | [-0.16, 2.30] |
|  | Other |  | -13.97 | 0.286 | [-39.6, 11.7] |  | 0.56 | 0.380 | [-0.69, 1.80] |
|  | Gender |  |  |  |  |  |  |  |  |
|  | Female |  | ref | ref | ref |  | ref | ref | ref |
|  | Male |  | -23.5 | 0.019 | [-42.99, -3.92] |  | 0.59 | 0.58 | [-0.39, 1.55] |
| Back-up compensatory | Age |  | 0.11 | 0.699 | [-0.45, 0.68] |  | 0.02 | 0.286 | [-0.2, 0.06] |
| (n = 240 HITs, 85 individuals) | VOR gain |  | -3.75 | 0.815 | [-35.12, 27.64] |  | 2.29 | 0.112 | [-0.53, 5.12] |
|  | Race |  |  |  |  |  |  |  |  |
|  | White |  | ref | ref | ref |  | ref | ref | ref |
|  | Black |  | -13.16 | 0.080 | [-27.88, 1.56] |  | 0.15 | 0.812 | [-1.05, 1.34] |
|  | Other |  | 1.46 | 0.819 | [-11.04, 13.96 ] |  | 0.65 | 0.153 | [-0.24, 1.55] |
|  | Gender |  |  |  |  |  |  |  |  |
|  | Female |  | ref | ref | ref |  | ref | ref | ref |
|  | Male |  | -12.17 | 0.018 | [-22.23, -2.11] |  | 0.67 | 0.087 | [0.10, 1.43] |

**Table 3. Relationship between VOR gain and overt saccades after limiting VOR gain < 1.2 and overt saccade latency to 170ms following the end of the HIT.** Relationship between VOR gain and 1) Saccade Latency, and 2) Saccade Amplitude for compensatory and back-up saccades. Significant results indicated by *.

|  |  |  | Saccade Latency | | |  | Saccade Amplitude | | |
| --- | --- | --- | --- | --- | --- | --- | --- | --- | --- |
| Saccade Type | Predictor Variables |  | β | p | 95% CI |  | β | p | 95% CI |
| Compensatory | Age |  | 0.27 | 0.387 | [-0.34, 0.88] |  | 0.03 | 0.000* | [0.013, 0.45] |
| (n = 1149 HITs, 175 individuals) | VOR gain |  | 21.24 | 0.121 | [-5.62, 48.11] |  | -4.68 | 0.000* | [-5.36, -4.01] |
|  | Race |  |  |  |  |  |  |  |  |
|  | White |  | ref | ref | ref |  | ref | ref | ref |
|  | Black |  | -4.99 | 0.415 | [-16.98, 7.00] |  | -0.002 | 0.990 | [-0.31, 0.31] |
|  | Other |  | -7.66 | 0.273 | [-21.37, 6.04] |  | 0.17 | 0.354 | [-0.19, 0.53] |
|  | Gender |  |  |  |  |  |  |  |  |
|  | Female |  | ref | ref | ref |  | ref | ref | ref |
|  | Male |  | -4.57 | 0.357 | [-14.29, -5.15] |  | 0.04 | 0.731 | [-0.21, 0.30] |
| Back-up compensatory | Age |  | -0.43 | 0.626 | [-2.15, 1.29] |  | -0.005 | 0.915 | [-0.096, 0.087] |
| (n = 72 HITs, 45 individuals) | VOR gain |  | 119.28 | 0.019 | [19.22, 219.34] |  | -3.07 | 0.011 | [-5.44, 0.70] |
|  | Race |  |  |  |  |  |  |  |  |
|  | White |  | ref | ref | ref |  | ref | ref | ref |
|  | Black |  | -28.68 | 0.069 | [-59.65, 2.28] |  | 0.42 | 0.612 | [-1.21, 2.06] |
|  | Other |  | -11.66 | 0.555 | [-50,34, 27.02 ] |  | -0.42 | 0.693 | [-2.48, 1.65] |
|  | Gender |  |  |  |  |  |  |  |  |
|  | Female |  | ref | ref | ref |  | ref | ref | ref |
|  | Male |  | -4.97 | 0.692 | [-29.53, -19.59] |  | 0.40 | 0.540 | [-0.88, 1.68] |
